# Spontaneous tension hemothorax in a severe COVID-19 patient receiving ECMO therapy: The other side of COVID-19-associated coagulopathy

**DOI:** 10.1016/j.rmcr.2022.101663

**Published:** 2022-05-06

**Authors:** Kanin Thammavaranucupt, Tanapat Tassaneyasin, Pongdhep Theerawit, Yuda Sutherasan, Pimwatana Pinsem, Supawadee Suppadungsuk, Nithita Nanthatanti, Suppachok Kirdlarp, Somnuek Sungkanuparph, Sirawat Srichatrapimuk

**Affiliations:** a Chakri Naruebodindra Medical Institute, Faculty of Medicine Ramathibodi Hospital, Mahidol University, Samut Prakan, Thailand; b Division of Critical Care Medicine, Department of Medicine, Faculty of Medicine Ramathibodi Hospital, Mahidol University, Bangkok, Thailand; c Division of Pulmonary and Pulmonary Critical Care Medicine, Department of Medicine, Faculty of Medicine Ramathibodi Hospital, Mahidol University, Bangkok, Thailand; d Department of Anesthesiology, Faculty of Medicine Ramathibodi Hospital, Mahidol University, Bangkok, Thailand

**Keywords:** COVID-19, ECMO, Hemothorax, Pulmonary embolism

## Abstract

As opposed to widely recognized Coronavirus Disease 2019 (COVID-19)-associated thrombotic events, the unusual but serious bleeding complications in COVID-19 patients are worth-mentioned. Here, we describe a 44-year-old man afflicted by COVID-19 pneumonia with acute respiratory distress syndrome (ARDS) and submassive pulmonary embolism. The patient's condition initially improved with the prescription of ECMO, tocilizumab, and hemoadsorption, however, he later developed spontaneous tension hemothorax, which is considered rare but devastating in the setting of COVID-19. While the exact pathogenesis of COVID-19-associated bleeding events remains poorly understood, we aim to highlight the other aspect of coagulation dysfunction potentially caused by COVID-19.

## 1 Introduction

Coronavirus Disease 2019 (COVID-19) caused by Severe Acute Respiratory Syndrome Coronavirus 2 (SARS-CoV-2) was first documented in December 2019 and was officially declared a pandemic by the World Health Organization (WHO) in March 2020. The disease has become a global health threat, with more than 270 million cases and 5.3 million deaths reported on 20^th^ December 2021 [1]. Presentations of COVID-19 range from asymptomatic to multiple organ failure. Acute respiratory distress syndrome (ARDS) is the most common complication of severe COVID-19. Other potentially life-threatening complications include secondary infections, thrombosis, pneumothorax, and bleeding [2].

Although spontaneous hemothorax has been recognized as a clinical entity for centuries, it is an uncommon complication in COVID-19 and has been rarely described in the medical literature. There is an urgent need to understand more about bleeding and thrombotic complications associated with COVID-19. We describe a COVID-19 patient with severe ARDS treated with venovenous extracorporeal membrane oxygenation (ECMO). The patient's course was complicated by pulmonary embolism and massive spontaneous hemothorax. We also discuss the probable causes and the management in this rare condition.

## 2 Case report

**Fig. 1:**
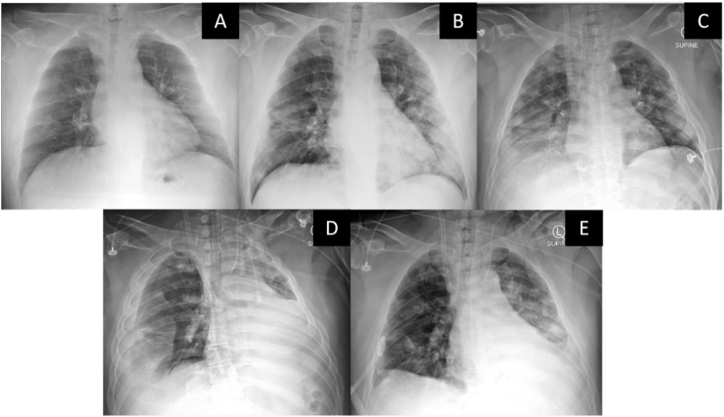
Serial portable chest radiographs of the patient. (A) Hospital day 1 (day 8 after the symptom onset). (B) Hospital day 8. (C) Hospital day 14 (ECMO day 1). (D) Hospital day 23 (ECMO day 10) – right hemothorax with left shift of mediastinum were noted and (E) after insertion of a percutaneous drainage tube to the right pleural space.

**Fig. 2:**
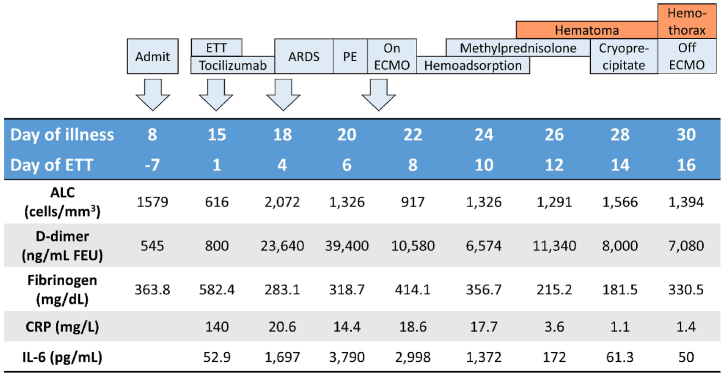
Levels of serum inflammatory markers, absolute lymphocyte count, d-dimer, and fibrinogen of the patient along the course of treatment; ALC: absolute lymphocyte count; ARDS: acute respiratory distress syndrome; CRP: c-reactive protein; ECMO: extracorporeal membrane oxygenation; ETT: endotracheal tube; FEU: fibrinogen-equivalent units; IL-6: interleukin-6; PE: pulmonary embolism.

A 44-year-old man with morbid obesity presented with a sore throat and productive cough for eight days and fever for four days. Physical examination showed body temperature of 37.9 °C, pulse rate of 86 beats/minute, respiratory rate (RR) of 22 breaths/minute, and blood pressure of 114/97 mmHg. His peripheral oxygen saturation was 95% while breathing ambient air. Chest radiography demonstrated ground glass opacities at lower lung fields (Fig. 1). Reverse transcription-polymerase chain reaction (RT-PCR) for SARS-CoV-2 from his nasopharyngeal swab was positive, fulfilling the diagnosis of COVID-19 pneumonia. Initial blood tests showed an absolute lymphocyte count of 1579/mm^3^, lactate dehydrogenase (LDH) of 234 U/L, and D-dimer of 545 ng/mL (Fig. 2). According to the national guideline for COVID-19 at the time, he was admitted (day 8 after the symptom onset) and placed on hydroxychloroquine, darunavir/ritonavir, azithromycin, and favipiravir.

**Fig. 3:**
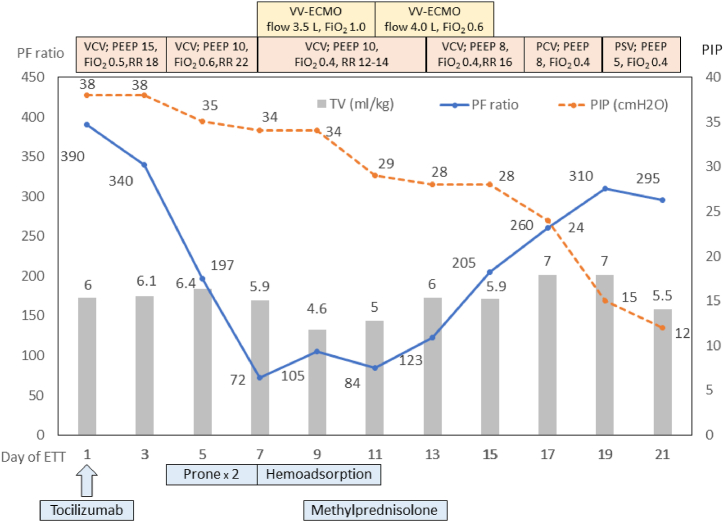
Clinical course of the patient and treatment received in the intensive care unit; PF–PaO_2_/FiO_2_; PIP: peak inspiratory pressure; VCV: volume control ventilation; PCV: pressure control ventilation; PSV: pressure support ventilation; ETT: endotracheal tube; PEEP: positive end expiratory pressure; RR: respiratory rate; vv-ECMO: veno-venous extracorporeal membrane oxygenation; TV: tidal volume.

On hospital day 8, he developed profound hypoxemia, and a chest radiograph revealed progression in patchy opacities at both lungs (Fig. 1). He was intubated, and the mechanical ventilator was initially set on volume control ventilation (VCV), with a tidal volume (TV) of 6 mL/kg predicted body weight, positive end-expiratory pressure (PEEP) of 15 cmH_2_O, RR of 18 breaths/min, and FiO_2_ of 0.5 (Fig. 3). A single dose of tocilizumab (640 mg intravenous) was given. However, his oxygenation gradually worsened, and his condition fulfilled ARDS criteria on hospital day 11 (ventilator day 4). His PaO_2_/FiO_2_ (PF) ratio decreased from 340 to 212, and the peak inspiratory pressure (PIP) increased to 45 cmH_2_O with a TV of 5.75 mL/kg predicted body weight, RR of 22 breaths/minute, PEEP of 10 cmH_2_O, and FiO_2_ of 0.6. Owing to the failure of lung-protective ventilation, prone positioning was employed (Fig. 3). The patient initially responded to this intervention. However, a progressive decline in the PF ratio was noted on the second attempt of prone positioning. Therefore, computed tomographic pulmonary angiography (CTPA) was done on hospital day 13, which revealed acute pulmonary embolism involving bifurcation of the main pulmonary artery, multiple bilateral segmental branches with small areas of bilateral basal lung infarction, and multiple ground-glass opacities scattered predominantly in bilateral peripheral lungs, without pulmonary artery aneurysm or pulmonary bleb. Therefore, a therapeutic dose of low molecular weight heparin (LMWH) was initiated. Bedside transthoracic echocardiography showed right ventricular dilatation, but left ventricular ejection fraction was preserved. A low-dose intravenous norepinephrine drip (0.05 mcg/kg/hr) was required to maintain his blood pressure.

Given the progressive deterioration of oxygenation despite prone positioning (Fig. 3) and the presence of right ventricular dysfunction, venovenous extracorporeal membrane oxygenation (vv-ECMO) was initiated on hospital day 14. The procedure was done without immediate complication. LMWH was replaced with intravenous unfractionated heparin. ECMO flow was at 3–4 L/min, a sweep of 2–3.5 L/min, and an FiO_2_ of 1.0. The FiO_2_ was reduced to 0.7 by ECMO day 4. Serial bedside transthoracic echocardiography of the patient revealed normalization of right ventricle size. During vv-ECMO therapy, activated clotting time (ACT) and activated partial thromboplastin time (aPTT) were maintained as standard practice. Platelet counts were above 80,000/mm^3^ at all times. On hospital day 14–17 (ECMO day 1–4), hemoperfusion with 5 HA-330 adsorbents was applied for cytokine removal. In addition, 1 mg/kg/day of methylprednisolone was administered on hospital day 16–20 (ECMO days 3–7). Decreased serum inflammatory markers and increased absolute lymphocyte counts were noted following days (Fig. 2). Vasopressor was discontinued on hospital day 16.

On hospital day 18 (ECMO day 5), a hematoma at the chest wall appeared and expanded from 5 × 5 cm to 33 x 12 cm. Even if ACT was in the expected range (180–240 seconds), these might be accounted for by previous prone position and coagulopathy. Cryoprecipitate was given, while intravenous heparin dose was decreased to maintain ACT around the lower limit. The chest wall hematoma and patient's lung compliance gradually improved, and the ECMO weaning process was initiated.

ECMO decannulation was planned on hospital day 23 (ECMO day 10), but patient unexpectedly became hypotensive and hypoxemic with need for high doses of norepinephrine. Chest radiograph showed a large right pleural effusion with a left shift of mediastinum (Fig. 1) suspicious for tension hemothorax. Heparin was discontinued, and immediate drainage tube was placed while simultaneously replacing blood. After 3 L of blood was drained, hypotension and hypoxemia rapidly improved. ECMO decannulation was then performed. On hospital day 24, CTPA revealed resolving pulmonary emboli, persistent patchy opacities, and a similar small are of basal lung infract as compared to prior. There was no evidence of active contrast extravasation. On hospital day 28, he was successfully extubated, followed by removal of his chest drainage on hospital day 32. He was restarted on his anticoagulation and discharged home on room air on hospital day 37.

## 3 Discussion

As the COVID-19 pandemic continues to grow, respiratory complications from COVID-19 such as ARDS, superimposed infections, pulmonary embolism, pneumothorax, and pneumomediastinum, are increasingly recognized [2]. While thrombotic complications in COVID-19 are quite well-recognized, patients are also at risk of bleeding complications. Spontaneous hemothorax has been reported as a rare complication of severe COVID-19. Various causes leading to this complication have been described. Necrotizing pneumonia was reported to account for the bleeding [3,4]. Vasculitis-like pulmonary artery aneurysms were also suggested to play a role [5]. Pulmonary embolism, a well-known complication of COVID-19, also predisposes patients for spontaneous hemothorax. Indeed, pulmonary infarction caused by emboli could lead to hemothorax in non-COVID-19 patients [6]. Additionally, a therapeutic anticoagulant used in this condition might also play a role. In our case, chest imaging before and after hemothorax showed thromboembolism in the pulmonary arteries without aneurysm or vascular leakage, and small areas of bilateral basal lung infarction.

With the application of ECMO in ARDS caused by COVID-19 currently growing, it is evident that bleeding complications in COVID-19 patients undergoing ECMO are not uncommon [7]. The underlying mechanism remains unclear. Disseminated intravascular coagulation (DIC) and acquired von Willebrand syndrome, as a result of extracorporeal circulation, have been proposed to account for this finding [8,9]. Additionally, anticoagulant use in patients on ECMO increases the bleeding risk, although it is indispensable for preventing thrombus formation in the ECMO circuit. Apart from iatrogenic aspects, severe COVID-19 itself could result in excessive fibrinolytic activation, culminating in enhanced-fibrinolytic-type DIC [[8], [9], [10]]. This condition is characterized by a strong activation of fibrinolysis with only subtle increase of plasminogen activating inhibitor, resulting in severe bleeding symptoms, as opposed to the prothrombotic state in DIC due to sepsis. Laboratory findings in enhanced-fibrinolytic-type DIC includes marked decrease in fibrinogen and elevation of fibrin degradation products (FDPs) out of proportion to d-dimer (increase FDP/d-dimer ratio). In our patient, laboratory results at the onset of chest wall hematoma followed by hemothorax (on hospital day 18–21) displayed a tendency of decreased fibrinogen and increased d-dimer levels, which were compatible with the features of enhanced-fibrinolytic-type DIC. This might contribute to the development of chest wall hematoma and spontaneous hemothorax, despite the appropriate ACT levels. Unfortunately, FDPs was not measured in our case. To summarize, beside traditional risk factors of bleeding complications including pulmonary embolism, anticoagulant and ECMO use in this patient, severe COVID-19 itself could contribute to the hemostatic defect.

The management of COVID-19 patients has not been clearly established. Massive hemothorax in patients receiving ECMO were reported to have a high mortality rate [11]. Suggested management in this condition includes emergent pleural drainage, video-assisted thoracoscopic surgery (VATS) and/or surgical thoracotomy, anticoagulant management, blood volume replacement, pleural epinephrine irrigation, and intravenous tranexamic acid [11]. In our patient, dose of heparin was adjusted and cryoprecipitate was given to raise the fibrionogen level at the onset of bleeding complication. It was also fortunate that he was ready for ECMO decannulation just before having hemothorax, and anticoagulant could thus be stopped at the time. Additionally, the intrathoracic bleeding improved without surgical operation, and he did not experience worsening of his pulmonary embolism thereafter. Taken together, COVID-19-associated bleeding complications are not uncommon, and might be as life-threatening as thrombotic complications [12]. In light of the widespread use of prophylactic anticoagulant in severe COVID-19 patients [13], further studies to determine bleeding risks in COVID-19 patients are warrant, to facilitate physician's decision in the management of anticoagulation therapy.

## 4 Conclusions

• Spontaneous hemothorax is a rare but crucial complication in COVID-19 patients. This complication might be brought about by both pathogenic alteration in respiratory system and systemic coagulopathy.
• In addition to thrombotic complications, severe COVID-19 together or not with pulmonary embolism, anticoagulant use, and ECMO therapy might result in significant bleeding complications.
• Risk and benefit of anticoagulant use in certain COVID-19 patients should be concerned.

## Authors’ contributions

Conceptualization: KT, SS, SSr. Data curation: SSr, NN. Formal analysis: KT, SSr. Methodology: PT, YS, PP, SSu, SK, NN. Project administration: KT, SSr. Visualization: KT, SSr, NN. Writing – original draft: KT, TT, SSr. Writing – review & editing: KT, SS, SSr.

KT: Kanin Thammavaranucupt.

SS: Somnuek Sungkanuparph.

SSr: Sirawat Srichatrapimuk.

SSu: Supawadee Suppadungsuk.

TT: Tanapat Tassaneyasin.

PT: Pongdhep Theerawit.

YS: Yuda Sutherasan.

PP: Pimwatana Pinsem.

NN: Nithita Nanthatanti.

SK: Suppachok Kirdlarp.

## Funding

This research did not receive any specific grant from funding agencies in the public, commercial, or not-for-profit sectors.

## Declaration of competing interest

No potential conflict of interest relevant to this article was reported.
